# Novel strategy of hold-and-drag clip closure with mantis-like claw for post-gastric endoscopic submucosal dissection defect of <30 mm

**DOI:** 10.1055/a-2213-4313

**Published:** 2023-12-21

**Authors:** Noriko Nishiyama, Takanori Matsui, Kaho Nakatani, Kazuhiro Kozuka, Naoya Tada, Tatsuo Yachida, Hideki Kobara

**Affiliations:** 1 Department of Gastroenterology and Neurology, Faculty of Medicine, Kagawa University, Miki, Japan

Endoscopic closure of post-endoscopic submucosal dissection (ESD) defects can reduce postoperative adverse events 1 . Innovative techniques such as use of an endoloop 2 , endoscopic ligation with O-ring closure 3 , and reopenable clip over the line method 4 have recently been developed; however, the complexity and time-consuming nature of such procedures remain problematic. We introduce a closure strategy using a new endoclip with a mantis-like claw (MANTIS Clip; Boston Scientific, Marlborough, Massachusetts, USA) ( Fig. 1 ) that enables a secure hold-and-drag maneuver and defect approximation in gastric post-ESD defects ( Fig. 2 ).

**Fig. 1:**
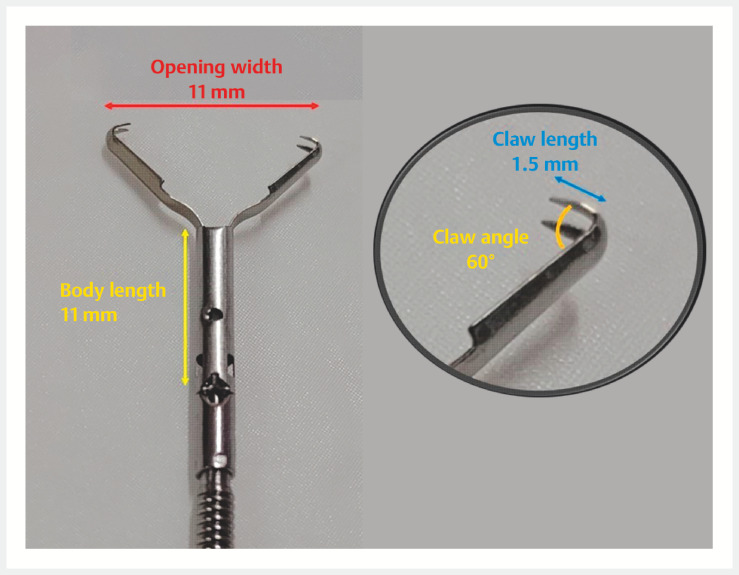
Details of the anchoring clip (MANTIS Clip; Boston Scientific, Marlborough, Massachusetts, USA). The clip contains TruGrip anchor prongs. The claw angle is 60° and the claw length is 1.5 mm. The length of the body is 11 mm and the opening width is 11 mm.

**Fig. 2:**
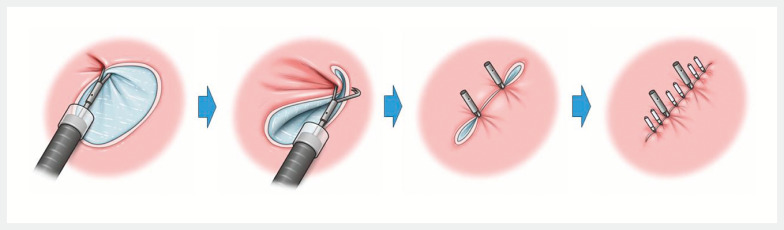
Schema of the closure procedure. Source: Davinch Medical Illustration Office.

A 56-year-old man presented with early gastric carcinoma in the lesser curvature of the mid-stomach. After standard ESD, a defect of 25 mm in diameter remained ( Fig. 3 **a** ). The defect was approximated using the MANTIS Clip by anchoring at the defect trisection points, followed by placement of standard clips between the MANTIS Clips ( Fig. 2 , Video 1 ).

**Fig. 3:**
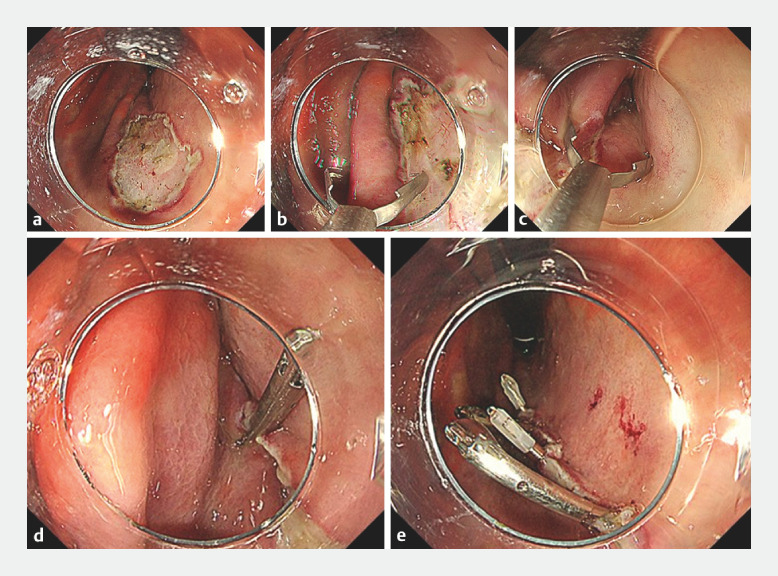
Defect closure. **a** After standard endoscopic submucosal dissection, a defect of 25 mm in diameter remained. **b** We held the edge of the defect at the distal trisection point and closed the clip. **c** The closed clip was dragged to the opposite edge of the defect. The clip was slowly reopened, without slipping from the anchored mucosa, and then reclosed with inclusion of the opposite edge of the defect. **d** After confirming that sufficient mucosa from both sides was grasped, the clip was deployed. **e** Finally, additional clips were deployed in the gaps between the two anchoring clips, and the whole defect was closed completely.

Use of new anchoring clips for closure of an artificial gastric defect. Source for graphical illustration: Davinch Medical Illustration Office.Video 1

First, one edge of the defect was anchored at the distal trisection point using the MANTIS Clip ( Fig. 2 , Fig. 3 **b** ). Second, the clip was dragged to the opposite edge of the defect. Third, when the clip was slowly reopened, the mantis-like claw maintained its anchor in the first edge without slipping, and approximated both edges of the defect ( Fig. 3 **c** ). After confirming successful grasping of both sides, the clip was deployed ( Fig. 3 **d** ). This procedure was repeated on the proximal side of the defect. Finally, additional standard clips (EZ Clip, HX-610–090L; Olympus, Tokyo, Japan) were deployed in the gaps between the two anchoring clips, and the whole defect was closed completely ( Fig. 3 **e** ). The procedure time was 7 minutes. The defect remained closed at 5 and 30 days post-procedure ( Fig. 4 ).

**Fig. 4:**
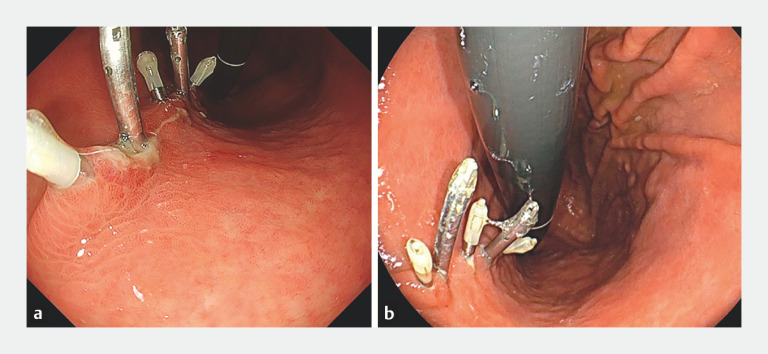
The closure and all clips remained intact postoperatively. **a** Assessment at 5 days. **b** Assessment at 30 days.

The efficacy of this technique for defects >30 mm is controversial because of submucosal dead space after mucosal closure 1 3 . However, this new anchoring clip may simplify and expedite gastric post-ESD defect closure.

Endoscopy_UCTN_Code_CCL_1AB_2AD_3AF

## Notes

Endoscopy E-Videos https://eref.thieme.de/e-videos
*E-Videos* is an open access online section of the journal *Endoscopy* , reporting on interesting cases and new techniques in gastroenterological endoscopy. All papers include a high-quality video and are published with a Creative Commons CC-BY license. Endoscopy E-Videos qualify for HINARI discounts and waivers and eligibility is automatically checked during the submission process. We grant 100% waivers to articles whose corresponding authors are based in Group A countries and 50% waivers to those who are based in Group B countries as classified by Research4Life (see: https://www.research4life.org/access/eligibility/ ). This section has its own submission website at https://mc.manuscriptcentral.com/e-videos .
